# Live human assessment of depth-dependent corneal displacements with swept-source optical coherence elastography

**DOI:** 10.1371/journal.pone.0209480

**Published:** 2018-12-28

**Authors:** Vinicius S. De Stefano, Matthew R. Ford, Ibrahim Seven, William J. Dupps

**Affiliations:** 1 Cole Eye Institute, Cleveland Clinic, Cleveland, OH, United States of America; 2 Dept. of Ophthalmology and Visual Sciences, Federal University of Sao Paulo, Sao Paulo, Brazil; 3 Dept. of Biomedical Engineering, Lerner Research Institute, Cleveland Clinic, Cleveland, OH, United States of America; 4 Dept. of Ophthalmology, Cleveland Clinic Lerner College of Medicine of CWRU, Cleveland, OH, United States of America; Cardiff University, UNITED KINGDOM

## Abstract

**Purpose:**

To assess depth-dependent corneal displacements in live normal subjects using optical coherence elastography (OCE).

**Methods:**

A corneal elastography method based on swept-source optical coherence tomography (OCT) was implemented in a clinical prototype. Low amplitude corneal deformation was produced during OCT imaging with a linear actuator-driven lens coupled to force transducers. A cross-correlation algorithm was applied to track frame-by-frame speckle displacement across horizontal meridian scans. Intra-measurement force and displacement data series were plotted against each other to produce local axial stiffness approximations, *k*, defined by the slope of a linear fit to the force/displacement data (ignoring non-axial contributions from corneal bending). Elastographic maps displaying local *k* values across the cornea were generated, and the ratio of mean axial stiffness approximations for adjacent anterior and posterior stromal regions, *k*_a_/*k*_p_, was calculated. Intraclass correlation coefficients (ICC) were used to estimate repeatability.

**Results:**

Seventeen eyes (ten subjects) were included in this prospective first-in-humans translational study. The ICC was 0.84. Graphs of force vs. displacement demonstrated that, for simultaneously acquired measurements involving the same applied force, anterior stromal displacements were lower (suggesting stiffer behavior) than posterior stromal displacements. Mean *k*_a_ was 0.016±0.004 g/mm and mean *k*_p_ was 0.014±0.004 g/mm, giving a mean *k*_a_/*k*_p_ ratio of 1.123±0.062.

**Conclusion:**

OCE is a clinically feasible, non-invasive corneal biomechanical characterization method capable of resolving depth-dependent differences in corneal deformation behavior. The anterior stroma demonstrated responses consistent with stiffer properties in compression than the posterior stroma, but to a degree that varied across normal eyes. The clinical capability to measure these differences has implications for assessing the biomechanical impact of corneal refractive surgeries and for ectasia risk screening applications.

## Introduction

The cornea is a unique tissue that effectively conserves its shape under a variety of physiological stresses while acting as a mechanical barrier to the ocular interior and maintaining transparency to visible light. Any variation in corneal geometry influences its principal function of focusing light to generate an image. Corneal ectatic diseases and refractive surgery procedures produce significant changes in the cornea’s structural and optical properties, and advances in the measurement of corneal biomechanical properties are needed to better characterize these responses, detect risk propensity, and predict individual responses to treatment.[1, 2]

Many factors contribute to the biomechanical homeostasis of the cornea. The collagen fibrillar architecture is a major determinant of corneal cohesive and tensile strength,[3–5] both of which are greater in the anterior stroma where significantly more oblique collagen branching and interweaving are present.[5–7] The non-fibrillar matrix components also contribute to the cornea’s biomechanical behavior and may play a role in ectatic diseases as well as the response to corneal crosslinking.[8, 9] Corneal hydration status impacts biomechanical property measurements,[10–12] as do environmental and pathological factors such as aging,[13] diabetes,[14] and eye-rubbing.[15] The cornea’s complex anisotropic composition and demonstrated heterogeneity in material properties argues for the development of measurement techniques that can resolve spatial property differences.[16] Commercially available devices such as the Ocular Response Analyzer (ORA, Reichert Technologies, Depew, NY, USA) and the Corvis ST (Oculus Optikgerate GmbH, Wetzlar, Germany) measure the corneal deformation response to a non-contact air puff stressor, and while both devices continue to advance our understanding of corneal biomechanical behavior in many clinical scenarios, neither is designed to assess spatially variant intracorneal property differences.[17]

Optical coherence elastography (OCE) is a conceptual extension of ultrasound elastography[18] in which a noninvasive low coherence interferometric imaging technique, optical coherence tomography (OCT), is used to image externally induced perturbations in a tissue.[19] This enables micrometer-scale spatial resolution and displacement sensitivity in various tissues, including the cornea.[20, 21] Several methods have been described to generate a perturbation and estimate displacement responses in the context of OCT elastography,[21–24] but to date, few *in vivo* corneal measurements have been reported in humans[25]. The motivation for the current study was to establish the feasibility of an OCT-based approach to clinical measurement of depth-dependent differences in live human subjects, to assess the preliminary reproducibility of the technique in the same clinical practice setting, and to assess the statistical significance of normative anterior-to-posterior property differences as a foundation for larger comparative studies.

## Methods

Seventeen normal eyes from ten human subjects were analyzed in this prospective translational pilot study. All the subjects were identified in a refractive surgery screening settings and were deemed suitable candidates for bilateral laser in situ keratomileusis (LASIK) surgery. An ophthalmologic exam was performed that included intraocular pressure (IOP) measurement using the Corvis and corneal tomography using the Pentacam HR (Oculus Optikgerate GmbH, Wetzlar, Germany). Exclusion criteria included any one of the following: a history of ocular disease, ocular trauma, previous eye surgery, central corneal thickness less than 500 μm, or tomographic signs of keratoconus or other corneal ectatic disease. The study was approved by the Cleveland Clinic Institutional Review Board (IRB #13–213) and all participants provided informed consent for research. All the procedures were conducted according to the Declaration of Helsinki, and the study was registered with clinicaltrials.gov (NCT03030755).

The OCE system consisted of a custom-built swept source (Santec HSL-20, Kamaki, Japan) OCT system with 9 μm axial coherence length in air and a spot size of approximately 20 μm in air with a scanning range of 15 mm by 15 mm laterally. The system operates at 1310nm central wavelength focused on the cornea and 15mW of optical power, which is below the ANSI Z136.1 recommended maximum permissible exposure. The OCT hardware was driven by a custom software suite created in house for elastography applications. The OCT imaging was performed using a previously described technique,[21] which consists of a fixed size scanning window with lateral oversampling (5 mm wide, 2 μm lateral sampling) to ensure accurate capture of the speckle pattern. Imaging was performed at a line rate of 100,000 A-scans/sec with no averaging.

**Fig 1:**
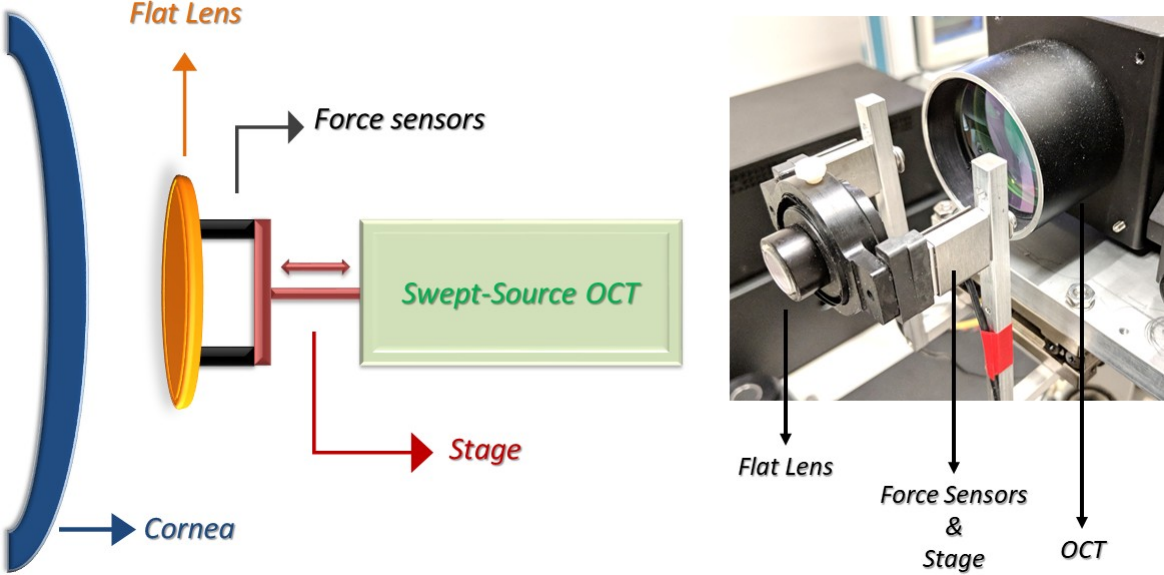
**Left**: OCT elastography system patient interface schematic diagram. **Right**: Photograph of the optical coherence elastography prototype highlighting the applanating apparatus (lens, force sensors and linear actuator stage).

The clinical prototype system and a schematic of the corneal interface elements are shown in Fig 1. The patient’s head was stabilized using a bite plate with a disposable, single-use cover to stabilize the skull and minimize motion. Proparacaine hydrochloride 0.5% drops were used before the procedure. A visual fixation target was used to facilitate repeatable alignment with the OCT sample arm. A flat glass plate 3mm thick was used to produce a compressive stress in these experiments. The compression plate is transparent to the OCT beam and was tilted slightly relative to the beam axis to prevent strong surface reflections. The plate was physically mounted to a computer-controlled linear actuator stage (ViX250IH, Parker Hannifin, Cleveland, OH), which was used to control its displacement towards the cornea. As the lens was axially displaced through a total range of 2 mm, one-hundred image frames were acquired across the horizontal meridian with the subject fixating a coaxial target over approximately 2.3 seconds. Two highly sensitive force sensors (Futek LSB200, Irvine, CA) incorporated in the system provided real-time measurement of the axial force component generated in response to the cornea’s contact with the compression plate. The two sensors were placed on opposite sides of the compression plate to provide data from both sides of the plate during compression and avoid disrupting the OCT beam.

Displacement tracking based on the OCT speckle pattern was performed in a frame-by-frame fashion as described in detail previously.[21] Corneal speckle is an optical feature of OCT that can be used as an intrinsic tissue fiducial marker for purposes of subsurface deformation tracking in response to a mechanical stimulus.[19] Custom software was used to compare two images from a temporal series using a window (22 x 22 pixels) that was taken from the first image and a series of windows from the second image near the origin of the first window. A cross-correlation algorithm was used to determine the maximum likelihood displacement vector for the speckle pattern within the corneal region of interest, as illustrated by the following equation:

$$C\left(x,y\right)=\frac{\sum_{x,y}\left[M\left(x,y\right)-\bar{M}\right]\left[N\left(x,y\right)-\bar{N}\right]}{{\left\{\sum_{x,y}{\left[M\left(x,y\right)-\bar{M}\right]}^{2}\sum_{x,y}{\left[N\left(x,y\right)-\bar{N}\right]}^{2}\right\}}^{0.5}}$$

The cross-correlation coefficient, *C*, is a measure of similarity of the speckle pattern between subsequent frames *N* and *M* and expresses the confidence that spatially displaced corneal speckle features represent the same tissue in a different location. In our previous work, signal to noise experiments were conducted and established a practical correlation coefficient threshold of 0.6 or higher as a tracking quality metric.[21] Regional displacement data with a C value below this threshold were excluded from analysis to reduce the likelihood of confounding from noise. Displacements were summed throughout the compression sequence to create a total displacement.

**Fig 2:**
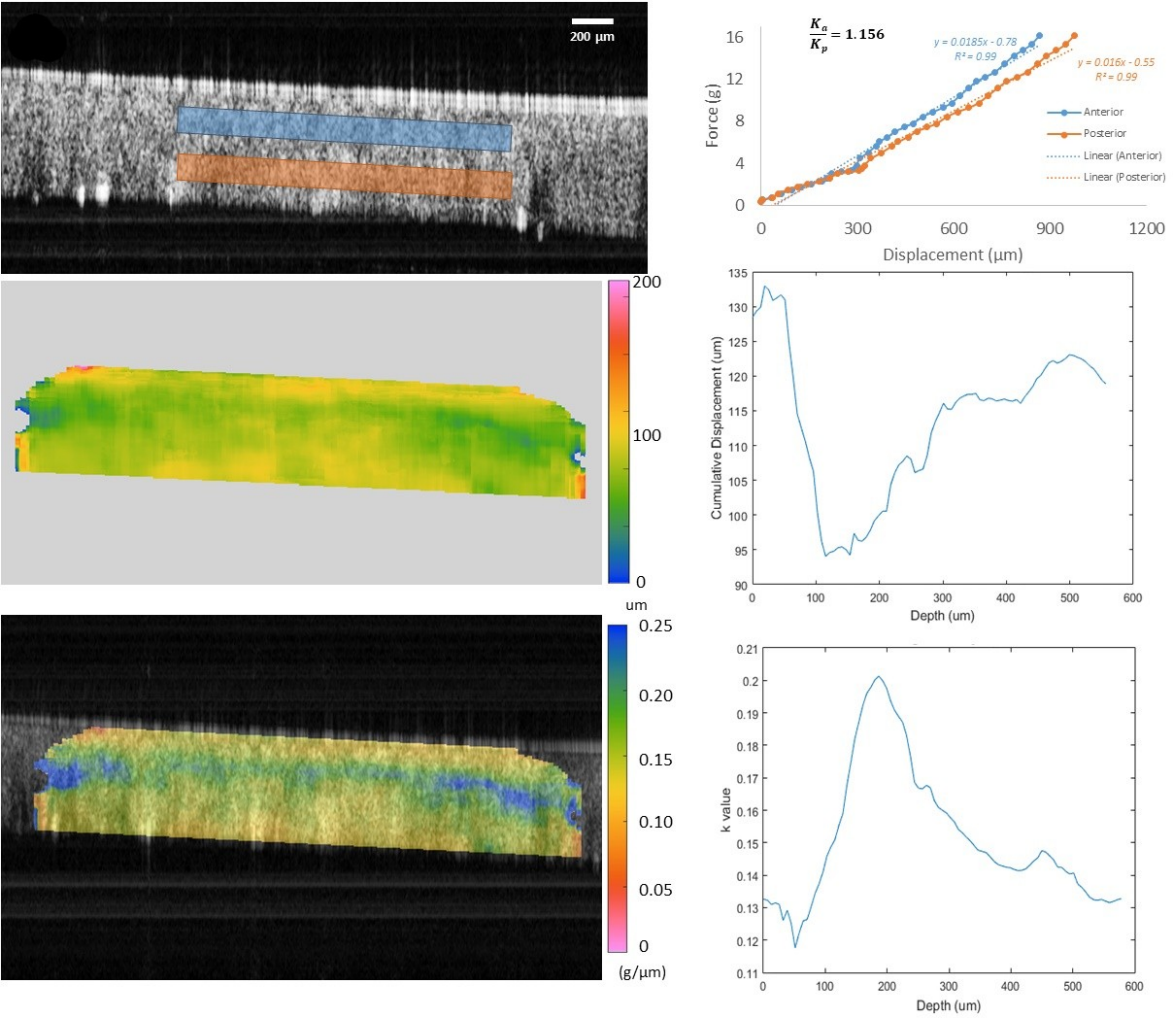
Example OCT elastography results for the right eye of patient one. **Top left:** Frame of the point of maximum compression. Anterior (blue box) and posterior (red box) stromal regions of interest were defined to generate the force/displacement relationships on the right. Note that the corneal epithelium was intact in all eyes (visible in top left frame) and was included in all analyses except the anterior/posterior stromal region comparisons. **Top right:** Force vs. displacement plot depicting differences in axial stiffness approximations for the anterior and posterior stromal regions. Higher slopes are consistent with stiffer behavior. **Middle Left**: Cumulative displacement map of the central cornea in microns. **Middle Right:** Depth-dependent cumulative displacement profile along a central 100um wide band (averaged laterally) through the depth of the cornea. **Bottom left:** Elastography map overlaid with the corneal image of the point of maximum compression presenting local values for the slope of the force (grams) vs. displacement (μm) relationship, an approximation of axial/compressive stiffness. Cooler colors indicate higher approximate stiffness corresponding to smaller displacement magnitudes than warmer colors. **Bottom right:** Depth-dependent k profile, representing the approximate stiffness behavior along a central 100um-wide band (averaged laterally) through the depth of the cornea.

After image extraction and displacement tracking results were obtained and spatially averaged, time-registered force data from the sensors and the axial component of the displacement vectors from cross-correlation analysis were analyzed to generate an axial stiffness analogue and cumulative displacement maps (Fig 2, top right panel). Anterior and posterior corneal regions of interest were defined, and each corresponded to a window of 1.6 mm horizontally by 150 μm axially (Fig 2, top left). The axial spacing between the anterior and posterior windows was approximately 120 μm. These analysis dimensions were chosen to provide meaningful representations of depth-dependent property differences while avoiding areas with sparse data due to thresholding by the anti-noise algorithm. The analysis focused on central regions of interest where complex effects from corneal curvature are less likely to complicate interpretation. Intra-measurement force and displacement data series were plotted against each other to produce an axial stiffness approximation, *k*, defined by:

$$k=\frac{f}{d}$$

where *f* is the measured force applied to the cornea by the compression plate and *d* is the local cumulative displacement of the cornea taken from the displacement map. While local intrastromal stresses are desirable for the calculation of a local stiffness metric, this information cannot be empirically measured for each point in the cornea, so the known surface force was used for calculating *k*. This working assumption is addressed further in the Discussion. Since the force/displacement relationship *k* evolves temporally across the compression sequence, this function was expressed as a slope value for statistical analysis purposes using a best-linear fit to the force and displacement data for the entire sequence (Fig 2, top right panel).

Elastographic maps displaying local *k* values across the cornea were generated (Fig 2, bottom left panel), and a continuous plot of local *k* values from anterior to posterior cornea was derived for each eye by averaging *k* values along 100um-wide lines at each axial depth location (Fig 2, lower right panel). To address prior evidence that anterior stromal stiffness is greater than posterior stiffness in normal human eyes, the ratio of average axial stiffness approximations for anterior and posterior stromal regions of interest, *k*_a_/*k*_p_, was also calculated and reported for each eye such that a value greater than 1 corresponds to greater anterior stiffness. Missing data points due to failed tracking as discussed above were replaced with a best-fit data point by a second order best-fit to the rest of the displacement sequence for that pixel. A minimum of five adjacent data points for the displacement sequence were required for the best fit replacement, otherwise the pixel was excluded from the analysis entirely. For color map generation, window sizes were optimized (approximately 80 μm axially by 120 μm laterally) to maximize visualization of local properties while maintaining adequate noise suppression.

To directly address the potential impact of curvature and IOP on the anterior/posterior k ratio in the context of the study data, linear regression analyses of clinical curvature (K_m_, the mean of the steep and flat keratometric values of the central cornea from Scheimpflug tomography) and clinical IOP to *k*_*a*_*/k*_*p*_ were performed (Minitab v18, State College, PA). As a preliminary assessment of measurement repeatability for this technique, 3 replicate measurements were obtained for each eye. The intraclass correlation coefficient (ICC) was calculated to determine the test-retest reliability.[26] Statistical analyses other than the regression analyses described above were performed with SPSS Statistics (v. 20, IBM Inc., Armonk, NY, USA).

### Donor eye and computational IOP sensitivity experiments

To perform preliminary analyses of the sensitivity of the anterior/posterior axial response ratio to physiological IOP variation, a donor-eye bench experiment and a computational study were performed. A finite element model of the *in vivo* measurement was developed replicating the clinical boundary conditions using one eye of the study. A corneoscleral whole globe model was generated based on a patient-specific corneal geometry of the eye from Pentacam (Oculus) and a generic sclera using custom meshing software (SpecifEye, OptoQuest, Cleveland, OH, USA) and the model was simulated using a commercial FE solver (Abaqus, Dassault Systemes, Waltham, MA, USA). Building on previously proposed microstructural approaches,[27, 28] the material model used for simulation of corneal stroma was a fiber reinforced model with an isotropic Neo-Hookean solid matrix. Pinsky et al.[27] proposed an analytical function that captures the main features of the anisotropic collagen fiber distribution. Angular integration of this analytical function was used at each integration point within the model to simulate the gradual change in collagen fiber orientation from the corneal center to its periphery.

In brief, the material model equations are as follows. The strain energy density function in the stroma is defined as:

$$W_{stroma}=W_{matrix}+\frac{1}{\int_{\alpha }\omega (r,\theta ,\varphi )}\int_{\alpha }\omega (r,\theta ,\varphi )\;W_{collagen}d\alpha$$

where *r*, *θ and φ* define the location and orientation of the fibers in polar coordinate system with fibril integration angle “θ” within the stroma, *α* represents the unit circle and W represents the strain density function. *ω* is the fiber distribution function defined by Pinsky et al.[27] The corneal isotropic matrix is modeled as a neo-Hookean material with c set to 0.04 MPa for the anterior stroma and linearly decaying to 0.02 MPa for the posterior stroma to represent a depth-dependent decrease in stromal biomechanical properties.[6, 29] The Cauchy stress of each collagen fiber is given by

$$\sigma_{collagen}=\frac{1}{I_{3}\lambda }\frac{\delta W_{collagen}}{\delta \lambda }b(r,\theta ,\varphi )⊗b(r,\theta ,\varphi )$$

where *I*_3_ is the third deviatoric Cauchy strain invariant, b is fiber orientation vector and *λ* is fiber stretch. Freed et al. defined the collagen fiber stress based on a 3D crimped helical fiber assumption in the appendix of their 2005 paper.[30] The three parameters of the helical collagen fiber are the initial normalized wavelength of the crimp set to 30.5, the initial normalized amplitude of the crimp set to 1.51, and the elastic modulus of the collagen fiber in the linear region set 32 MPa. The fibril orientation and fiber-component elastic modulus remained consistent throughout the corneal depth in this sensitivity analysis. The material properties were optimized using an inverse analysis to match previously published corneal inflation and tensile data.[31]

The flat lens was simulated as a rigid body since the elastic modulus of the lens is significantly higher than the cornea and the sclera. A frictionless contact was defined between the posterior surface of the flat lens and the anterior surface of the cornea. The simulation was performed in multiple steps. First, the stress-free configuration of the cornea was computed using the method described in the aforementioned reports. Following this computation, the stress-free configuration was copied in the contact model and the eye was loaded with a specific IOP. Finally, the lens compression was simulated in the presence of IOP loading. This sequence was repeated for 10, 15, and 20 mm-Hg IOP values, then displacement data from anterior and posterior stromal regions of interests were extracted from the finite element model and presented in surface force/regional displacement relationships analogous to those generated for human subjects.

OCE elastography measurements at the same IOP levels were also performed on one normal 48-hour post-mortem human donor eye globe from a 65-year-old male donor with no previous ocular surgical history. The IOP of the globe was controlled with a height adjustable saline bag inserted into vitreous cavity through a 16-gauge needle. Pressure at the height of the globe was measured with an in-line pressure transducer connected to a digital pressure monitor. The measurement protocol was repeated for IOPs of 10, 15, and 20 mmHg, and force and displacement data were recorded for each repetition as described in the elastography protocol detailed above.

## Results

Descriptive clinical and tomographic data for patients included in this study are presented in Table 1. No eyes were excluded from the study based on the criteria described in the previous section. A representative example of an OCE force/displacement measurement is displayed in Fig 2. A pattern of lower displacement magnitudes in the anterior cornea compared to the posterior cornea is visible, suggestive of higher relative stiffness in the anterior stroma. The number of available temporal data frames varies between datasets due to slight variations in the location of the patients’ eye when the scan is initiated. While the compression sequence is not varied, the point of initial lens contact with the corneal apex within the automated compression sequence does vary slightly. The mean number of temporally distinct frames available was 29 ± 6.5 frames per imaging sequence. The cross-correlation algorithm yielded an average correlation coefficient of 0.68 ± 0.02, which meets the threshold for accurate displacement tracking described in the Methods and determined previously in signal-to-noise sensitivity experiments.[21]

**Table 1:** Demographic and tomographic data for each subject.

Subject	Age	Gender	Eye	Spherical Equivalent Manifest Refraction	IOP (mmHg)	Pentacam			
						Kmean (D)	Kmax (D)	TPCT (μm)	BAD-D
1	29	Male	OD	-4.38	15.5	41.6	42.5	529	0.53
			OS	-2.63	14.5	42.0	42.7	532	0.52
2	30	Male	OD	0.75	17.0	39.3	39.7	590	0.30
			OS	0.50	16.0	39.4	39.9	603	0.24
3	60	Female	OD	3.00	15.5	42.2	43.0	543	1.05
			OS	4.13	14.0	42.0	42.4	530	1.21
4	34	Female	OD	-0.50	13.0	42.0	43.4	535	1.42
			OS	-0.75	13.0	42.2	43.6	534	1.87
5	32	Female	OD	-4.13	17.0	41.8	43.0	535	1.10
			OS	-3.38	16.0	42.2	43.3	538	1.47
6	26	Male	OS	-1.00	19.0	42.0	43.0	559	0.55
7	32	Female	OS	-2.88	14.5	42.5	43.2	509	1.27
8	31	Female	OD	-6.00	15.0	46.6	47.1	542	1.04
			OS	-6.25	15.0	46.7	47.2	537	1.36
9	23	Male	OS	-0.25	18.5	44.7	45.4	591	1.26
10	39	Male	OD	-3.63	12.5	45.3	46.0	536	1.93
			OS	-3.63	13.5	45.5	46.4	535	1.81

IOP = intraocular pressure; Km = mean simulated keratometry value; Kmax = maximum point curvature value; TPCT = thinnest point of corneal thickness; BAD-D = Belin-Ambrosio enhanced ectasia total deviation (D) score

Calculation of the ICC using the single measures approach yielded a value of 0.84 (95% CI: 0.60–0.94). Every patient had at least two useable scans. When three reasonable scans were available, the two highest quality measurements (assessed by the number of frames in which the cornea is being compressed and signal-to-noise ratio analysis) were selected for calculation of the ICC.

The calculated *k*_a_, *k*_p_ and *k*_a_/*k*_p_ values for each measurement sequence are displayed in Table 2 along with the R^2^ value of each linear fit to the force/displacement data. The very high degree of linear correlation suggests an overwhelmingly linear force/displacement relationship for the anterior and posterior stroma across the loading regime used for these measurements. The *k*_a_/*k*_p_ values averaged 1.123 ± 0.062, indicating that, on average, the anterior cornea displaced less than the posterior cornea during simultaneous perturbations for all the tested eyes. Similar anterior to posterior differences are appreciable in the color maps of the displacement vs. force curves (Fig 2, lower left panel) which show lower displacement magnitudes towards the anterior stroma and the corneal periphery (cooler colors) and higher displacements in the posterior stroma (warmer colors). The behavior is also observable in the depth-dependent cumulative displacement and *k* profile graphs, which demonstrate a decrease in displacement (increase in *k)* below the epithelium followed by a reversal in the deeper stroma.

**Table 2:** Approximation of axial stiffness from the slope term of the force/displacement relationship for anterior and posterior corneal stroma.

Subject	Eye	R^2^_a_	R^2^_p_	*k*_a_	*k*_p_	*k*_a_/*k*_p_
1	OD	0.99	0.99	0.0185	0.0160	1.156
	OS	0.99	0.99	0.0156	0.0129	1.209
2	OD	0.99	0.99	0.0180	0.0161	1.118
	OS	0.97	0.98	0.0173	0.0137	1.263
3	OD	0.99	0.99	0.0165	0.0144	1.146
	OS	0.99	0.99	0.0180	0.0157	1.146
4	OD	0.99	0.99	0.0183	0.0153	1.196
	OS	0.98	0.98	0.0182	0.0161	1.130
5	OD	0.97	0.96	0.0121	0.0120	1.008
	OS	0.98	0.99	0.0179	0.0168	1.065
6	OS	0.98	0.99	0.0238	0.0227	1.048
7	OS	0.95	0.95	0.0129	0.0114	1.132
8	OD	0.93	0.94	0.0110	0.0101	1.089
	OS	0.98	0.99	0.0105	0.0096	1.094
9	OS	0.97	0.98	0.0176	0.0161	1.093
10	OD	0.96	0.97	0.0092	0.0084	1.095
	OS	0.99	0.99	0.0113	0.0102	1.108
mean		0.98	0.98	0.0157	0.0140	1.123
standard deviation		0.02	0.02	0.0039	0.0035	0.062

R^2^_a_ and R^2^_p_: R^2^ of the linear fit of the slope for the anterior (a) and posterior (p) force/displacement relationship. k_a_ and k_p_: slope of the force/displacement function for anterior (a) and posterior (p) corneal stromal regions.

**Fig 3:**
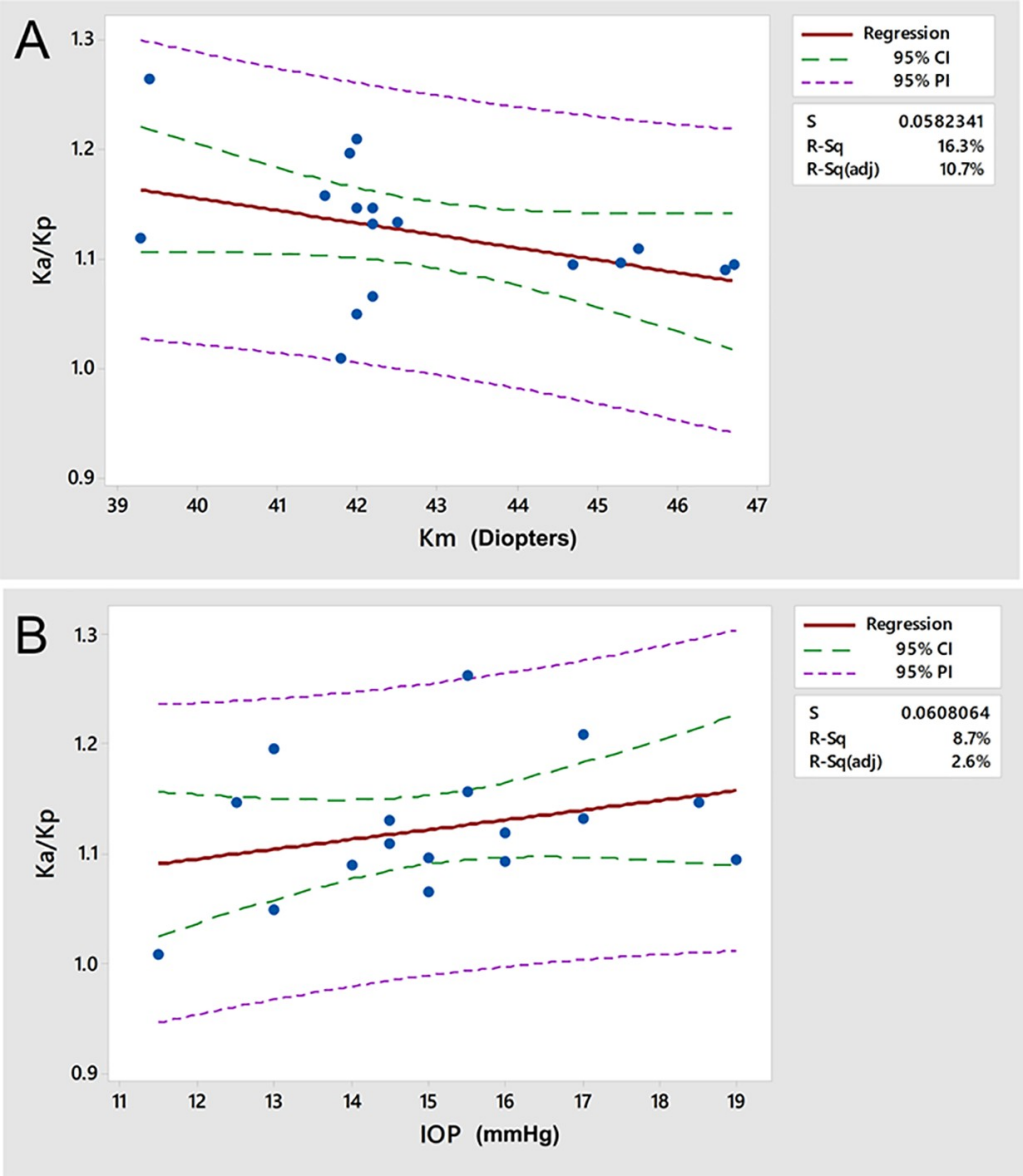
Linear regression results between k_a_/k_p_ (the anterior/posterior stromal response ratio) and **A**) the mean anterior corneal curvature and **B**) IOP for all eyes. Despite a trend toward lower with increasing corneal curvature, neither correlation was statistically significant. S = standard error, R-sq = coefficient of variation, CI = confidence interval, PI = prediction interval.

The actuator moves a total distance of 2 mm including pre-contact movement, which leads to a slightly different number of data points acquired per patient. The total measured force is used as a normalization factor for the specific compressive force generated against the IOP in each measurement sequence. Regression analyses between *k*_*a*_*/k*_*p*_ and the potential covariants mean anterior corneal curvature and IOP demonstrated no significant linear relationships (R^2^ = 8.7%, p = 0.1, and R^2^ = 16.7%, p = 0.3, respectively). Scatter plots and linear regression models for each analysis are presented in Fig 3.

**Fig 4:**
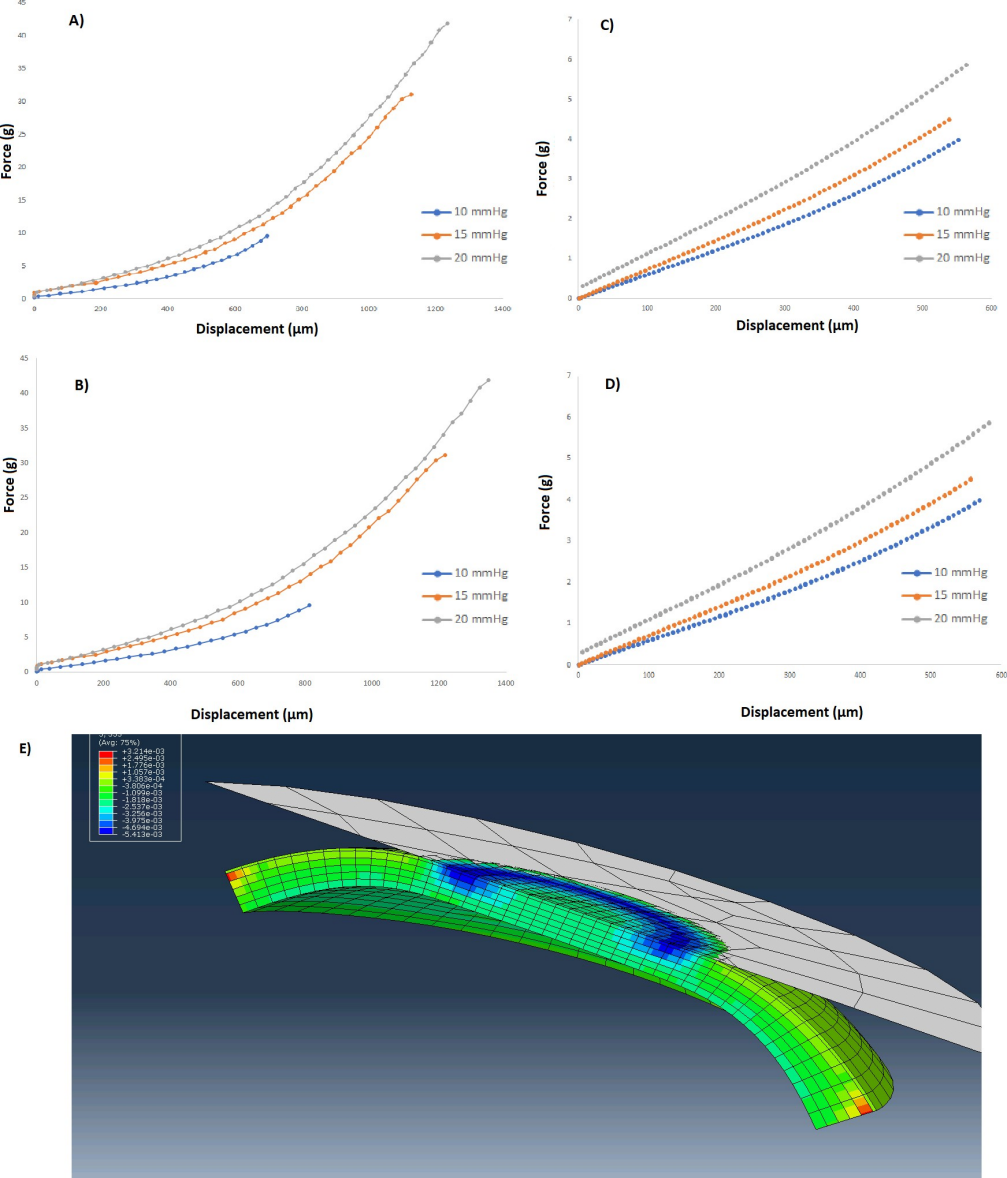
System sensitivity to the intra-eye IOP variability. Anterior (A), and posterior (B) force/displacement curves from donor eye experiment with IOP values of 10, 15, and 20 mmHg. Anterior (C) and posterior (D) force/displacement curves from in silico experiment with 10, 15, and 20 mmHg IOPs. E: Axial corneal stresses under compression from in-silico study at IOP = 15mmHg.

Table 3 demonstrates the force vs. displacement curves from the globe experiment and the computational exercise. Both *in silico* and bench studies showed that *k*_*a*_*/k*_*p*_ ratio varied minimally and, in the case of the donor eye, minimally and non-systematically in response to varying IOPs. Table 3 shows *k*_*a*_*/k*_*p*_ values for 10, 15, and 20 mmHg values for the donor eye and in-silico experiments. Fig 4 shows the axial stresses in the cornea under axial perturbation (IOP = 15mmHg) as well as force/displacement curves of anterior and posterior cornea from donor and in-silico perturbations with different IOPs.

**Table 3:** Axial stiffness ratio between anterior and posterior corneal regions in response to varying intraocular pressures.

IOP (mmHg)	Simulated *k*_*a*_*/k*_*p*_	Donor-eye *k*_*a*_*/k*_*p*_
10	1.032	1.144
15	1.033	1.087
20	1.034	1.105
*Mean*	*1*.*033*	*1*.*112*
*Standard Deviation*	*0*.*001*	*0*.*029*

IOP: intraocular pressure; k_a_ and k_p_: slope of the force/displacement function for anterior (a) and posterior (p) corneal stromal regions.

## Discussion

This study presents the first *in vivo* human application of a compression-based optical coherence elastography method for spatially sensitive corneal biomechanical measurement. The clinical capability to measure depth-dependent differences in corneal biomechanical response has important implications for assessing the biomechanical impact of corneal refractive surgeries and identifying deviations from normal patterns in refractive surgery screening. For example, the rationale for the structural advantage of intrastromal lamellar corneal refractive procedures such as small incision lenticule extraction (SMILE) over flap-based procedures such as LASIK relies on the assumption that the anterior corneal stroma has greater elastic strength than the posterior cornea.[32, 33] While prior ultrastructural work and biomechanical testing in *ex vivo* human corneas support the consistent presence of an elasticity gradient in the normal cornea,[28, 29, 34, 35] data from live human subjects has been reported for only one eye to date using Brillouin scattering.[36] We anticipated some patient-specific variation in absolute and relative depth-dependent properties across normal subjects, and characterizing this variation is important for better understanding patient-specific surgical risk. Even with this variation, the ratio of anterior to posterior properties was greater than 1 in every eye measured in this normal subject series. In contrast, we expect that the loss of collagen interweaving noted in the region of Bowman layer in keratoconus explants[4] may manifest as early biomechanical weakening of the anterior stroma. Such changes would conceivably alter the normal depth-dependent property profile and possibly offer utility as an early biomarker for ectasia risk. A clinical study is underway to further address this hypothesis, and this study serves as an important precursor for establishing a normal range of anterior-to-posterior gradients for powering such comparisons.

Several studies in ex vivo human explants have demonstrated evidence of an anterior-to-posterior corneal stromal property gradient. Kohlhaas *et al*. performed extensiometry on anterior and posterior corneal lamellar specimens from 5 human donor corneas and found Young’s modulus to be higher in the anterior stroma.[37] Randleman et al. demonstrated depth-dependent decreases in cohesive tensile strength in 20 human donor strips measured at 3 different stromal depths.[29] More recently, Mikula *et al*. also found higher anterior values for elastic modulus in 6 donor eyes using acoustic radiation force elasticity microscopy at two target depths.[35] A nondestructive OCT elastography approach using micro-puff air perturbations as elastic wave generators has been described for mapping wave velocities in agar phantoms and *ex vivo* porcine and rabbit tissue,[24, 38] but application of the method to the live human cornea has not yet been reported. Dorronsoro *et al* similarly combined a noncontact air tonometer and spectral domain OCT that was applied to one eye of a human subject,[39] but only the corneal surface deformation dynamics were monitored. As referenced above, Scarcelli and Yun reported the first *in vivo* spatially resolved measurement of corneal biomechanical properties in a single human eye using Brillouin microscopy and observed a depth-dependent decline in Brillouin shift within the cornea.[36] The current report demonstrates the presence of a depth-dependent elastic property gradient for the first time in a series of normal living human subjects and presents the first spatial biomechanical property clinical data derived from an OCT-based approach.

There are several important advantages to assessing corneal biomechanical behavior using the *in vivo* methodology described here. Relative anterior and posterior compressive behaviors were measured in live, healthy human corneas over a short measurement duration exposed to the same experimental conditions. This is an important intrinsic control given that previous *ex vivo* studies with OCE and other measurement methods show significant dependence of biomechanical properties on experimental hydration conditions.[11, 12] Also, the axial displacement behaviors of the anterior and posterior cornea (and thus the resulting derivations of *k*_*a*_*/k*_*p*_) were obtained during the same compressive regime, at the same time, under the same anterior surface loading force, and with no destructive sectioning of the tissue or removal of the cornea from its native boundary conditions. The measurements, which indicated a higher stiffness behavior in the anterior stroma in every subject (*k*_*a*_*/k*_*p*_ >1), are therefore not subject to common sources of error associated with testing excised samples at different times, under different loads (including intraocular pressure), or under different hydration conditions.

While there are several advantages to the measurement regime, an applanating perturbation imposed on the convex curvature of the cornea produces potentially complex displacements related to rotation and dependencies on corneal geometry and IOP. As described in the Methods section, the displacement analysis was confined to the axial component of the displacement vectors and concentrated on the central cornea. Both of these strategies should markedly reduce the impact of rotational deformations on the resulting axial stiffness approximations, but it important to note that the stiffness approximation ignores non-axial contributions from corneal bending and makes a simplifying assumption of constant force through the depth of the cornea To more directly assess the possibility that corneal geometry and IOP played confounding roles in the interpretation of *k*_*a*_*/k*_*p*_ as an approximation of relative anterior/posterior properties in this dataset, regression analyses were performed that demonstrated no significant linear relationships between *k*_*a*_*/k*_*p*_ and either IOP or anterior curvature.

While the current results are consistent with prior work in terms of the presence and direction of a corneal property gradient, the mean anterior-to-posterior stiffness ratio was slightly lower than most prior results from *ex vivo* tissue. However, the aforementioned controls in this series and fundamental differences in the perturbation regimes and tissue states between studies impact the comparability of the results. For example, testing of lamellar cohesive force[29] at a specific corneal depth emphasizes the resistance to peeling force at a single intrastromal interface, whereas indentation of femtosecond laser-excised lenticules from different corneal depths captures axial force/displacement data for samples that are removed from their native boundary conditions and loading forces.[6] These testing methods both illustrate important differences in related but distinctly different constitutive mechanical properties across the human corneal stroma. The latter study employing axial indentation testing is particularly germane to the current study because both studies involve axial compression testing regimes. Although axial displacement behavior is measured perpendicular to the plane of the collagen lamellae, the relevance of this mode of measurement is supported by the work of Winkler *et al*. which 1) demonstrated the presence of significant depth-dependent differences in stromal compressive resistance and 2) observed strong correlations between axial modulus differences and the degree of collagen fiber intertwining.[6] Differences in regional tissue properties may be greater in stromal samples that are separated from their neighboring tissues than in whole corneas still coupled to their native surroundings since neighboring tissue properties confine deformation to some degree. It should also be noted that the 2 regions of interest that were defined in the current study to produce the *k* ratio (Fig 2) do not represent the entire corneal thickness, so the finding that posterior stromal displacement was greater than anterior stromal displacement should not be interpreted as requiring an increase in corneal thickness. The finite element simulation punctuates this point by producing an analogous difference in displacements without requiring an increase in corneal thickness.

In the current study, a practical emphasis was placed on the analysis of axial displacements. Lateral displacement tracking was performed simultaneously and is integral to the cross-correlation code, but preliminary analysis of the data suggests that 1) the vast majority of displacement behavior under this compressive regime is captured by the axial component of the displacement vector and 2) additional registration strategies will likely be required to address the influence of bulk displacement from horizontal saccades on lateral displacement vectors. A more complete representation of out-of-plane and regional behavior during a compression series is of great interest, particularly in applications where detection of localized biomechanical abnormalities is desired. Previous pilot studies done in our lab in porcine eyes, in which a three-dimensional displacement tracking was feasible even at current scanning speeds, suggested that lateral and out-of-plane displacements were minor contributors to the total displacement vector in the setting of an applanating perturbation. Although the current clinical protocol emphasized reducing total patient scan time and maximizing the quality of the single-meridian data, three-dimensional, multi-region capture for *in vivo* scanning is being developed.

Since the current elastography method incorporates continuous force measurements at the corneal surface during the perturbation and imaging sequence, it is possible to relate surface forces to measured intracorneal displacements and arrive at an analytic approximation of local compressive elastic moduli. However, mechanical noise from movement of the force transducer cable during patient alignment produced artefactual force data in some of our early measurements, requiring different offsetting points in each scan and preventing accurate absolute force measurements at the point of initial corneal loading. By focusing on the question of relative anterior/posterior properties in this series, the identical, simultaneously acquired surface force terms are neutralized in the division and the comparison remains fully dependent on relative displacement behavior. System changes were implemented to avoid aberrant force data at the point of initial contact, and future datasets will support analysis of differences in absolute *k* value between eyes.

As mentioned in the Methods, exact local intrastromal stresses are not known and cannot be readily measured *in vivo*. Finite element analysis (FEA) can be used to estimate them, but the derived stresses depend on model assumptions, mesh characteristics and other variables. As explained in the previous paragraph, the current study emphasizes a relative measure of stiffness driven by local displacement behavior. But to better understand the likely impact of the simplifying assumption that corneal stress is uniform through the cornea in relating displacement data to stiffness, we replicated the in vivo measurement scheme in FEA. These simulations suggest that axial stresses generated by the lens perturbation under the clinical loading regime are similar in the analyzed regions of interest in the presence of a depth-variant modulus. To the extent that depth-dependent compressive stresses are similar between these regions and the axial surface force approximates the axial stresses within the tissue, the *k*_*a*_*/k*_*p*_ ratio more closely reflects a true stiffness ratio. In practice, it is still possible that displacements might not be uniform in axial space even in the setting of material properties that are invariant with depth. The adjunctive FEA analyses support the conclusion that the measurement method is appropriately sensitive to regional differences in elastic behavior. Efforts are underway to use patient-specific OCE displacement and force data in inverse finite element solutions of local elastic properties and to represent displacement in the form of strain for more direct biomechanical inference.

Absolute *k* values are likely to be sensitive to IOP due to the dependence of the surface reaction force on the eye’s internal fluid pressure, but the measurement and the force/displacement formula also incorporate a direct applanation pressure as a normalizing factor for contextualizing the displacement behavior. Therefore, for the purposes of the current results, the differential displacement properties of the anterior and posterior cornea should be less sensitive to IOP since the measurements are relative and simultaneously acquired. In the donor eye and *in silico* IOP sensitivity analyses, the dependence of the *k*_*a*_*/k*_*p*_ ratio on physiological variations IOP was negligible and at least an order of magnitude lower than the inter-subject variability of the measure (Table 3 and Table 2, respectively). The *k*_*a*_*/k*_*p*_ ratio cancels out the force values from the relative measure by division, leaving a purely displacement-based anterior-posterior response comparison. These results, in combination with the post-hoc regression analysis described earlier indicating no correlation between *k*_*a*_*/k*_*p*_ and IOP from same-day noncontact tonometry in the clinical subjects, suggest that IOP is not a significant or systematic confounder of the anterior/posterior response ratio. Inverse FEA is being explored as a comprehensive strategy for obtaining local elastic modulus estimates from displacement/force relationships since a more complete representation of contributing structural factors (IOP, corneal geometry, and corneal boundary conditions) can be achieved.[27, 40–42]

## References

[1] Kling S, Hafezi F. Corneal biomechanics—a review. *Ophthalmic Physiol Opt* 2017; 37: 240–252. 10.1111/opo.12345 28125860

[2] Dupps WJ, Wilson SE. Biomechanics and wound healing in the cornea. *Exp Eye Res* 2006; 83: 709–720. 10.1016/j.exer.2006.03.015 16720023 PMC2691611

[3] Seiler T, Matallana M, Sendler S, Bende T. Does Bowman’s layer determine the biomechanical properties of the cornea? Refract Corneal Surg 1992; 8: 139–42. 1591208

[4] Morishige N, Wahlert AJ, Kenney MC, Brown DJ, Kawamoto K, Chikama T-I, et al Second-harmonic imaging microscopy of normal human and keratoconus cornea. *Invest Ophthalmol Vis Sci* 2007; 48: 1087–94. 10.1167/iovs.06-1177 17325150 PMC1894888

[5] Smolek MK, McCarey BE. Interlamellar adhesive strength in human eyebank corneas. *Investig Ophthalmol Vis Sci* 1990; 31: 1087–1095. 2354912

[6] Winkler M, Chai D, Kriling S, Nien CJ, Brown DJ, Jester B, et al Nonlinear optical macroscopic assessment of 3-D corneal collagen organization and axial biomechanics. *Investig Ophthalmol Vis Sci* 2011; 52: 8818–8827. 22003117 10.1167/iovs.11-8070 PMC3230904

[7] Boote C, Elsheikh A, Kassem W, Kamma-Lorger CS, Hocking PM, White N, et al The influence of lamellar orientation on corneal material behavior: biomechanical and structural changes in an avian corneal disorder. *Investig Opthalmology Vis Sci* 2011; 52: 1243–1251. 10.1167/iovs.10-5962 PMC3101698 21051696

[8] Borcherding MS, Blacik LJ, Sittig RA, Bizzell JW, Breen M, Weinstein HG. Proteoglycans and collagen fibre organization in human corneoscleral tissue. *Exp Eye Res* 1975; 21: 59–70. 124659 10.1016/0014-4835(75)90057-3

[9] Quantock AJ, Young RD, Akama TO. Structural and biochemical aspects of keratan sulphate in the cornea. *Cell Mol Life Sci* 2010; 67: 891–906. 10.1007/s00018-009-0228-7 20213925 PMC11115788

[10] Kling S, Marcos S. Effect of hydration state and storage media on corneal biomechanical response from in vitro inflation tests. *J Refract Surg* 2013; 29: 490–497. 10.3928/1081597X-20130617-08 23820232

[11] Ford MR, Roy AS, Rollins AM, Dupps WJ. Serial biomechanical comparison of edematous, normal, and collagen crosslinked human donor corneas using optical coherence elastography. *J Cataract Refract Surg* 2014; 40: 1041–1047. 10.1016/j.jcrs.2014.03.017 24767794 PMC4035481

[12] Hatami-Marbini H, Etebu E. Hydration dependent biomechanical properties of the corneal stroma. *Exp Eye Res* 2013; 116: 47–54. 10.1016/j.exer.2013.07.016 23891861

[13] Knox Cartwright NE, Tyrer JR, Marshall J. Age-related differences in the elasticity of the human cornea. *Investig Opthalmology Vis Sci* 2011; 52: 4324–4329. 10.1167/iovs.09-4798 20847118

[14] Kotecha A, Oddone F, Sinapis C, Elsheikh A, Sinapis D, Sinapis A, et al Corneal biomechanical characteristics in patients with diabetes mellitus. *J Cataract Refract Surg* 2010; 36: 1822–8. 10.1016/j.jcrs.2010.08.027 21029887

[15] Bawazeer AM, Hodge WG, Lorimer B. Atopy and keratoconus: a multivariate analysis. *Br J Ophthalmol* 2000; 84: 834–836. 10.1136/bjo.84.8.834 10906086 PMC1723585

[16] Singh M, Li J, Han Z, Wu C, Aglyamov SR, Twa MD, et al Investigating Elastic Anisotropy of the Porcine Cornea as a Function of Intraocular Pressure With Optical Coherence Elastography. *J Refract Surg* 2016; 32: 562–567. 10.3928/1081597X-20160520-01 27505317 PMC5409833

[17] De Stefano VS, Dupps WJ. Biomechanical diagnostics of the cornea. *Int Ophthalmol Clin* 2017; 57: 75–86. 10.1097/IIO.0000000000000172 PMC5463580 28590282

[18] Ophir J, Cespedes I, Ponnekanti H, Yazdi Y, Li X. Elastography: a quantitative method for imaging the elasticity of biological tissues. *Ultrason Imaging* 1991; 13: 111–134. 10.1177/016173469101300201 1858217

[19] Schmitt J. OCT elastography: imaging microscopic deformation and strain of tissue. *Opt Express* 1998; 3: 199–211. 19384362 10.1364/oe.3.000199

[20] Larin K V., Sampson DD. Optical coherence elastography–OCT at work in tissue biomechanics. *Biomed Opt Express* 2017; 8: 1172–1202. 10.1364/BOE.8.001172 28271011 PMC5330567

[21] Ford MR, Dupps WJ, Rollins AM, Roy AS, Hu Z. Method for optical coherence elastography of the cornea. *J Biomed Opt* 2011; 16: 0160051–0160057. 10.1117/1.3526701 PMC3041813 21280911

[22] Dupps WJ, Netto M V, Herekar S, Krueger RR. Surface wave elastometry of the cornea in porcine and human donor eyes. *J Refract Surg* 2007; 23: 66–75. 17269246 10.3928/1081-597x-20070101-11 PMC2075088

[23] Wang S, Larin K V, Li J, Vantipalli S, Manapuram RK, Aglyamov S, et al A focused air-pulse system for optical-coherence-tomography-based measurements of tissue elasticity. *Laser Phys Lett* 2013; 10: 1–6. 10.1088/1612-2011/10/7/075605 PMC5969524 29805349

[24] Singh M, Li J, Vantipalli S, Wang S, Han Z, Nair A, et al Noncontact elastic wave imaging optical coherence elastography for evaluating changes in corneal elasticity due to crosslinking. *IEEE J Sel Top Quantum Electron* 2016; 22: 266–276. 10.1109/JSTQE.2015.2510293 PMC4990138 27547022

[25] Alonso-Caneiro D, Karnowski K, Kaluzny BJ, Kowalczyk A, Wojtkowski M. Assessment of corneal dynamics with high-speed swept source Optical Coherence Tomography combined with an air puff system. *Opt Express* 2011; 19: 14188–14199. 10.1364/OE.19.014188 21934782

[26] Koo TK, Li MY. A guideline of selecting and reporting intraclass correlation coefficients for reliability research. *J Chiropr Med* 2016; 15: 155–63. 10.1016/j.jcm.2016.02.012 27330520 PMC4913118

[27] Pinsky PM, van der Heide D, Chernyak D. Computational modeling of mechanical anisotropy in the cornea and sclera. *J Cataract Refract Surg* 2005; 31: 136–45. 10.1016/j.jcrs.2004.10.048 15721706

[28] Pandolfi A, Holzapfel GA. Three-dimensional modeling and computational analysis of the human cornea considering distributed collagen fibril orientations. *J Biomech Eng* 2008; 130: 061006 10.1115/1.2982251 19045535

[29] Randleman JB, Dawson DG, Grossniklaus HE, McCarey BE, Edelhauser HF. Depth-dependent cohesive tensile strength in human donor corneas: implications for refractive surgery. *J Refract Surg* 2008; 24: S85–S89. 18269156 10.3928/1081597X-20080101-15

[30] Freed AD, Einstein DR, Vesely I. Invariant formulation for dispersed transverse isotropy in aortic heart valves: an efficient means for modeling fiber splay. *Biomech Model Mechanobiol* 2005; 4: 100–17. 10.1007/s10237-005-0069-8 16133588

[31] Elsheikh A, Wang D, Brown M, Rama P, Campanelli M, Pye D. Assessment of corneal biomechanical properties and their variation with age. *Curr Eye Res* 2007; 32: 11–9. 10.1080/02713680601077145 17364730

[32] Sinha Roy A, Dupps WJ, Roberts CJ. Comparison of biomechanical effects of small-incision lenticule extraction and laser in situ keratomileusis: finite-element analysis. *J Cataract Refract Surg* 2014; 40: 971–80. 10.1016/j.jcrs.2013.08.065 24857440 PMC6030688

[33] Reinstein DZ, Archer TJ, Randleman JB. Mathematical model to compare the relative tensile strength of the cornea after PRK, LASIK, and small incision lenticule extraction. J Refract Surg 2013; 29: 454–460. 10.3928/1081597X-20130617-03 23820227

[34] Mikula E, Hollman K, Chai D, Jester J V., Juhasz T. Measurement of corneal elasticity with an acoustic radiation force elasticity microscope. *Ultrasound Med Biol* 2014; 40: 1671–1679. 10.1016/j.ultrasmedbio.2013.11.009 24726798 PMC5919192

[35] Mikula ER, Jester J V, Juhasz T. Measurement of an elasticity map in the human cornea. *Investig Ophthalmol Vis Sci* 2016; 57: 3282–3286. 27327584 10.1167/iovs.15-18248 PMC4961063

[36] Scarcelli G, Yun SH. In vivo Brillouin optical microscopy of the human eye. *Opt Express* 2012; 20: 9197–9202. 10.1364/OE.20.009197 22513631 PMC3500092

[37] Kohlhaas M, Spoerl E, Schilde T, Unger G, Wittig C, Pillunat LE. Biomechanical evidence of the distribution of cross-links in corneas treated with riboflavin and ultraviolet A light. *J Cataract Refract Surg* 2006; 32: 279–83. 10.1016/j.jcrs.2005.12.092 16565005

[38] Wang S, Larin K V. Noncontact depth-resolved micro-scale optical coherence elastography of the cornea. *Biomed Opt Express* 2014; 5: 3807 10.1364/BOE.5.003807 25426312 PMC4242019

[39] Dorronsoro C, Pascual D, Pérez-Merino P, Kling S, Marcos S. Dynamic OCT measurement of corneal deformation by an air puff in normal and cross-linked corneas. *Biomed Opt Express* 2012; 3: 473 10.1364/BOE.3.000473 22435096 PMC3296536

[40] Sinha Roy A, Dupps WJ. Effects of altered corneal stiffness on native and postoperative LASIK corneal biomechanical behavior: A whole-eye finite element analysis. J Refract Surg 2009; 25: 875–87. 10.3928/1081597X-20090917-09 19835328

[41] Sinha Roy A, Dupps WJ. Patient-specific modeling of corneal refractive surgery outcomes and inverse estimation of elastic property changes. *J Biomech Eng* 2011; 133: 011002 10.1115/1.4002934 21186892

[42] Seven I, Vahdati A, De Stefano VS, Krueger RR, Dupps WJ. Comparison of patient-specific computational modeling predictions and clinical outcomes of LASIK for myopia. *Investig Opthalmology Vis Sci* 2016; 57: 6287–6297. 10.1167/iovs.16-19948 PMC5119490 27893094

